# The influence of power dynamics and trust on multidisciplinary collaboration: a qualitative case study of type 2 diabetes mellitus

**DOI:** 10.1186/1472-6963-12-63

**Published:** 2012-03-13

**Authors:** Julie McDonald, Rohan Jayasuriya, Mark Fort Harris

**Affiliations:** 1 Centre for Primary Health Care and Equity, University of New South Wales, Sydney, Australia; 2 School of Public Health and Community Medicine, University of New South Wales, Sydney, Australia

## Abstract

**Background:**

Ongoing care for chronic conditions such as diabetes is best provided by a range of health professionals working together. There are challenges in achieving this where collaboration crosses organisational and sector boundaries. The aim of this article is to explore the influence of power dynamics and trust on collaboration between health professionals involved in the management of diabetes and their impact on patient experiences.

**Methods:**

A qualitative case study conducted in a rural city in Australia. Forty five health service providers from nineteen organisations (including fee-for-service practices and block funded public sector services) and eight patients from two services were purposively recruited. Data was collected through semi-structured interviews that were audio-taped and transcribed. A thematic analysis approach was used using a two-level coding scheme and cross-case comparisons.

**Results:**

Three themes emerged in relation to power dynamics between health professionals: their use of power to protect their autonomy, power dynamics between private and public sector providers, and reducing their dependency on other health professionals to maintain their power. Despite the intention of government policies to support more shared decision-making, there is little evidence that this is happening. The major trust themes related to role perceptions, demonstrated competence, and the importance of good communication for the development of trust over time. The interaction between trust and role perceptions went beyond understanding each other's roles and professional identity. The level of trust related to the acceptance of each other's roles. The delivery of primary and community-based health services that crosses organisational boundaries adds a layer of complexity to interprofessional relationships. The roles of and role boundaries between and within professional groups and services are changing. The uncertainty and vulnerability associated with these changes has affected the level of trust and mistrust.

**Conclusions:**

Collaboration across organisational boundaries remains challenging. Power dynamics and trust affect the strategic choices made by each health professional about whether to collaborate, with whom, and to what level. These decisions directly influenced patient experiences. Unlike the difficulties in shifting the balance of power in interprofessional relationships, trust and respect can be fostered through a mix of interventions aimed at building personal relationships and establishing agreed rules that govern collaborative care and that are perceived as fair.

## Background

The prevalence of chronic disease in the Australian population is rising. Diabetes is a major cause of mortality, morbidity and disability and an important risk factor for several other chronic diseases [1]. Diabetes is the second most frequent chronic condition managed in Australian general practice and the most frequent reason for referral to other health care providers [2], reinforcing the importance of the primary health care sector in diabetes care. General practice guidelines for Type 2 Diabetes Mellitus (T2DM) highlight the need for access to well coordinated health care from a range of medical and allied health care professionals, including GPs, medical specialists, diabetes educators, dieticians, optometrists, and podiatrists [3]. Referrals to allied health professionals such as diabetes educators or dieticians are low, even among patients who are overweight or obese [4], indicating that there is a need to improve collaborative care across professional and organisational boundaries.

Previous research has examined collaboration between GPs and other community-based health professionals. Disagreements and conflicts over roles and role boundaries and a lack of shared decision-making suggest that issues of power and authority are important factors in these relationships and influence the patterns of collaboration [5-7]. Trust and respect are also important enablers of collaboration and mistrust and perceived lack of respect are barriers [5,8-10].

Resource dependency theory and transaction cost analysis have been used to explore collaboration amongst healthcare organisations [11,12]. These theories propose that collaboration is a function of the need for resources. The need for resources creates uncertainties and dependencies for organisations that they strive to reduce while maintaining their autonomy and pursuing their interests. Resource dependency theory emphasises the importance of resources to the organisation and contends that their concentration by other organisations determines the nature of the interdependency and power relations [13,14]. Transaction cost analysis emphasises the role of governance arrangements to regulate relationships and address the uncertainties about the behaviour of others, particularly their trustworthiness [15]. Van de Ven and Walker [16] argue that, in the health context, relationships established for the purpose of referring clients tend to develop on a case-by-case basis, are less formalised, and rely more on personal knowledge and trust among interacting parties.

Power has been described according to: (a) who has formal authority to make decisions and who controls the resources; and (b) who has less tangible aspects of symbolic power or the ability to control ideas and meaning [17]. Power differences based on unequal professional status are an example of the latter. Hardy and Phillips [17] argue that it is the distribution of these tangible and intangible resources in interorganisational relationships which determines the strategies of engagement; namely the choice between strategies based on cooperation or conflict. The same could also be said for interprofessional relationships where the sources of power differentials, including the broader social, cultural and professional systems, produce and reinforce the power imbalances [18]. In the hierarchy of health professions, doctors have traditionally defended their professional autonomy and independence and professional status in their relationships with other health care workers. As Hudson found, these 'turf wars' maybe intra-professional as well as inter-professional [19]. Other research points to the situational context of power, with relationships between health professionals in hospital settings mediated by the exercise of medical dominance as opposed to the use of more collegiate approaches found in community settings [20].

Trust is a way of handling uncertainty and risk in the delivery of collaborative healthcare that crosses organisational and professional boundaries. It involves the expectation that other parties will behave in ways that are predictable and fair, that they are competent and will refrain from opportunistic behaviour [21]. As a feature of interprofessional relationships, trust is often related to concepts of competence, professional identify and respect [18,22]. Trust is also viewed as an earned characteristic that develops over time [22]. Where opportunities to develop personal relationships are limited, the use of rules and norms to govern behaviour can substitute for interpersonal trust [23]. Referral arrangements between health professionals are an example of such norms. However, there is a paradox: while rules and norms can substitute for trust, the transaction costs associated with these strategies could be reduced if there was mutual trust between the parties [21].

The concepts of power and trust from the perspective of resource dependency theory and transaction cost analysis provide a lens for investigating interprofessional relationships among primary and other community-based health services. To our knowledge this perspective has not been used to examine interprofessional relationships that cross organisational boundaries. We use the example of collaboration for the management of T2DM to address the following research question: how do power dynamics and trust influence interprofessional relationships, and how do these factors impact on the experiences of patients receiving care from multiple providers.

### Setting and context

The structure of the Australian health system sets some important challenges for achieving more collaborative service delivery. Two levels of government are responsible for policy, planning and service delivery and there is a lack of integration between their various initiatives [24]. The primary health care sector comprises a diverse range of health professions, disciplines and practitioners working in private and public sector organisations of varying size and complexity and under different funding arrangements. GPs and allied health professionals in private practice operate on a fee-for-service basis, and public sector health professionals are funded through block funding arrangements. These structural differences make it difficult to collaborate as there are few opportunities for communication and for the development of personal relationships.

Policy initiatives by the Commonwealth government to improve the management of T2DM are part of a broader focus aimed at improving chronic disease management in Australia. Since 1999/2000 financial incentives have been introduced for GPs to support more systematic and coordinated care. In 2005 with the introduction of the Chronic Disease Management (CDM) program, the financial incentives were extended to allied health professionals to support access to more comprehensive and affordable care for patients referred by a GP. GPs are required to develop a multidisciplinary care (MDC) plan which identifies the care goals and allied health professionals involved in the delivery of services to achieve these goals. The care plan is shared with the patient and the relevant allied health professionals who are also required to report back to the referring GP. The agreement is known as a Team Care Arrangement (TCA). The allied health incentives are restricted to practitioners working in private practice and a total of five occasions of service per year. No additional funding has been provided for public sector health professionals, although they can be included in the MDC plan and TCA. Financial incentives and other policy initiatives to support practice nurses have seen a significant increase in their numbers, with approximately 57% of general practices employing at least one practice nurse [25]. Their roles include prevention and chronic disease management, quality and integration of patient care, and liaison with external providers. These initiatives have not, however, been matched by policies regarding their relationships and roles as regards other community-based nurses who have similar chronic care roles. Both groups have developed in isolation from one another.

## Methods

The study used a qualitative case study approach to explore the experiences and perceptions of participants involved in providing or receiving care for T2DM. It was approved by a New South Wales Health Department accredited ethics committee (HNEHREC 08/05/21/4.09) and participants gave full and informed consent.

### Selection and recruitment

The research was conducted in a mid-sized inland rural city with a population of approximately 40,000. Overweight and obesity rates are higher than average as is the prevalence of diabetes. Health services comprise primary, secondary and tertiary services provided through public and private sector organisations (or practices), including the region's public sector tertiary referral hospital and community health services (the area health service). Given the city's distance from other major centres, most people receive most of their diabetes related health care locally. These factors meant that the case study was well bounded and provided an opportunity to explore collaboration in an environment that was sufficiently, but not overly, complex.

To be included in the study, organisations needed to have a major role in providing primary and community-based health services for adults with T2DM and/or a role in diabetes prevention. In addition to the allied health professional categories identified in the introduction, other allied health professionals who were identified by local informants as having a role in diabetes were pharmacists, physiotherapists and fitness instructors, community nurses (wound management) and Aboriginal workers (healthy lifestyle programs). Organisations that provided comparable services were sampled according to size and then by convenience.

Organisations were invited to nominate at least one health practitioner representing each relevant profession. The four services that were part of the area health service were treated as separate organisations as they delivered distinct services. Thirty five practitioners representing 13 professions/equivalent from 19 organisations participated (see Table 1). Table 2 shows the broad profile of the sample in relation to the age range and length of employment in the organisation from which they were recruited. Based on information included in a local health services directory, the gender balance for most categories was equivalent to that found in the local population of health professionals. An additional 10 interviews were held with managers from the area health service, the division of general practice (the local GP support organisation) and the community centre.

**Table 1:** Summary of participating health professionals

Health professional type	Number	Health professional type	Number
*Private sector*		*Public sector (Area Health Service)*	

Dietician	1	Aboriginal health education officer	1

General practitioner	5	Community nurse	3

Practice nurse	2	Diabetes educator (nurse)	2

Medical specialist	3	Dietician	7

Optometrist	2	Fitness instructor	1

Pharmacist	1	*Non-government sector*	

Physiotherapist	1	Aboriginal community worker	1

Podiatrist	5	*TOTAL*	*35*

**Table 2:** Summary characteristics of participating health professionals

Provider	Gender		Age range				Length of employment in service				
	
	M	F	21-30	31-40	41-50	51+	> 1 yr	1-5	6-10	11-15	16+
Allied health professionals	4	14	7	5	6	0	2	9	3	2	2

GPs	3	2	0	1	1	3	0	2	2	0	1

Medical Specialists	2	1	0	1	0	2	1	0	0	0	2

Nurses (including diabetes educators)	0	7	0	1	3	3	0	3	1	0	3

Other practitioners	2	0	0	0	2	0	0	2	0	0	0

*Total*	*11*	*24*	*7*	*8*	*12*	*8*	*3*	*16*	*6*	*2*	*8*

Eight adult patients were purposively recruited from three of the participating organisations: two general practices which were running diabetes clinics, and the diabetes centre which had a major role in the provision of treatment and patient education services. Inclusion criteria for patients required that they were currently receiving diabetes care from the respective organisation and at least one other locally based health professional. Recruitment was performed by each organisation following a written protocol. Patients were excluded if they had multiple complications, a recent hospital admission or other factors which made them ineligible to participate in a telephone interview. Two females and six males between the ages of 51-76 participated who had been diagnosed with T2DM for less than one year to 14 years. Two patients were being managed by diet alone, six on tablets, and the remaining two were on a combination of tablets and insulin.

### Data collection

Semi-structured interviews were conducted by the principal researcher (PR) between November 2008 and September 2010. The purpose of the practitioner interviews was to gain in-depth understanding of their perceptions and experiences in collaborating across organisational and professional boundaries. Six pilot interviews informed the development of an interview guide, but were not included in the analysis. The guide covered the following areas: (a) background information on the profile of practice/service/organisation and of the interviewee; and (b) exploratory questions about their collaboration with outside organisations and health practitioners in relation to diabetes prevention and care: who, for what, why, and how it worked; satisfaction; benefits and drawbacks; factors that helped/hindered; to what extent they saw themselves as part of a network/team of health professionals, and who else was part of this network. The guide was used as a check list to ensure each of these topic areas was covered at some stage during the interview. Interviewees were encouraged to describe and reflect on their experiences and perceptions in some detail and through the use of examples. Enlisting the commitment of health professionals working under fee-for-service arrangements meant that there was limited opportunity for prolonged engagement with them in the research process, and each participant was interviewed only once, with times ranging from 30-45 minutes. Most interviews were held face-to-face during a 2-week site visit in late 2008. Two group feedback sessions (each lasting two hours) on the preliminary findings were held in late 2009 presented opportunities to collect further data.

The purpose of patient interviews was to explore their experiences of, and satisfaction with, receiving care from health professionals working in different organisations, particularly regarding coordination of care. Coordination was defined as continuity or the degree to which care was experienced as coherent, connected and consistent with their needs [26]. Two pilot interviews informed the development of an interview guide, but were not included in the analysis.

### Analysis

Interviews and feedback sessions were audio-taped and transcribed verbatim for thematic analysis of the content, with 55% transcribed by an independent transcription service, and the remainder by the PR who also coded all transcripts. A selection of transcripts of different health professionals was also coded by a co-investigator to identify additional insights. Data management was assisted by the use of a computer program (NVivo 8). The analyses of the provider and patient interviews were undertaken separately but followed a similar process and was guided by three generic analysis strategies involved in qualitative research described by Creswell [27]:148-154: (a) preparing and organising the data, (b) reducing the data to themes through a process of coding and condensing, and (c) representing the data in figures and tables. A two-level coding scheme was used, starting with a provisional list of codes based on selected concepts identified in the literature, with new codes added based on the data [28]. The coding structure was revised during the analysis through reflecting on the research and consulting the literature. Initially the codes were a mix of descriptive categories and higher order concepts [29]:82. Through an iterative process of coding and condensing the data, recurring themes emerged. Creswell describes thematic analysis as "aggregating the information into larger clusters of ideas" [27]:244. This process involved: 1) displaying the coding structure in visual maps to explore how the codes could be grouped together under topic areas or themes and to identify possible relationships; and 2) constructing thematic tables to assist with interpreting the data, and to compare findings between participants within each data source. Negative case analysis was employed to look for alternative explanations and disconfirming evidence [27]:208. Rigour was enhanced through the following validation strategies described in the literature [27]:207-208: (a) feedback sessions on early findings to health professional participants (a form of member checking), (b) peer debriefing sessions with other researchers to test and critique the emerging themes, findings and patterns, and (c) maintaining a reflexive journal throughout the research process which documented personal and analytic reflections and research decisions.

## Findings

The findings are structured around the themes that emerged that related to: (a) the two relationship factors of power dynamics and trust, and (b) the patient experiences of the impact of these interprofessional relationships. Quotes are used selectively to give voice to the participants and to illustrate the meanings.

### Use of power by health professionals to protect their autonomy

Government policies have extended the gate-keeping role of GPs to allied health professionals, and hence their access to resources. Increased GP referrals through TCA provide allied health professionals with an additional source of clients and income.

"We see more Medicare side of things now where patients with diabetes who are having difficulty with regular exercise as a consequence of osteo-arthritic changes in their lower limbs." (Physiotherapist)

The power of GPs over allied health professionals, however, is limited. Patient referrals and TCA involve a low level of professional interdependency and involve few risks to the professional autonomy of allied health professionals as they have other sources of income (e.g. fee-paying self referrals) over which GPs have little influence. Despite these constraints to the authority of GPs, they implicitly asserted their medical dominance in other ways. They rarely developed MDC plans in consultation with other providers, there was very little two-way communication and the information that they shared varied considerably.

" If we get a very brief care plan it's very hard to sort out exactly what the doctor's and patient's expectations are from the consultation. If you've got a good care plan that outlines exactly what the goals are, it's so much easier." (Podiatrist 5)

### Power dynamics between private and public sector providers

Collaboration between GPs and public sector health professionals was more complicated and both groups strived to maintain their authority over what they considered their area of expertise. GPs had little formal authority or responsibility for the management or care coordination of patients in public sector health services. Most public sector nurses and allied health practitioners relied on referrals from other practitioners within the area health service and client self-referrals. In this context, the gate-keeping role and medical dominance of GPs were circumvented, and the professional status of non-medical practitioners protected as indicated by the following quote:

" The literature from the diabetes educators' association says that if you are newly diagnosed it is still best practice to be educated by a diabetes educator and dietician. When discharging clients, we make it clear to them that if they feel they need to return, they don't have to come back through a doctor's referral. They can refer themselves." (Diabetes educator 1)

Practice nurses together with GPs were running diabetes clinics in two of the larger general practices in the study. These nurses carried out much of the routine diabetes monitoring and patient education that was traditionally undertaken by diabetes educators (who were nurses by profession). The concerns about the consistency of care being provided, as illustrated in the following quote, could be a reflection of threats to the professional standing of the diabetes educators posed by less trained practitioners taking on these roles.

"There was concern from the diabetes educators: how are GPs and practice nurses delivering this information? If they're going to be supporting newly diagnosed diabetics, .....are they giving them consistent messages or are the diabetes educators going to get the patients three months down the track and have to address the inconsistency of messages." (Manager, Division of General Practice)

### Health professionals maintain their power by reducing their dependency on selected health professionals

Health professionals minimised the threats to their autonomy and independence through two major approaches: (a) choosing partners with whom they had cooperative relationships, (b) reducing their collaboration with health professionals outside their organisation. The introduction of financial subsidies for some allied health care gave GPs a broader range of affordable referral choices outside the public sector, which they preferred to use. The relationship was mutually beneficial to both parties and involved little conflict or threats to existing power relations.

" (GPs) should work with a practice nurse to manage diabetes.. and refer patients to private sector dieticians, podiatrists, and optometrists. So we can do it within in the private sector that way." (GP 1)

Access to medical practitioners working at the area health service meant that public sector nurses and allied health professionals could limit their collaboration with GPs, and they used a mix of strategies to achieve this: applying their own criteria to gauge the priority of GP referrals, maintaining self-referral relationships with patients and declining to participate in TCAs, for example:

*"There has been some resistance....The GPs have wanted us to sign off as a second care giver, but we haven't ... for some reason we weren't happy about it ... I don't know whether you'd call it professional jealousy or what." (Community nurse 1)*.

Larger general practices reduced their need to collaborate with external practitioners by developing their own skills and capacity to provide diabetes care. Some general practices also entered into co-location arrangements with allied health professionals, although the latter remained independent practitioners and maintained their own patient records. Through the opportunities for informal communication, co-location contributed to the development and maintenance of cooperative relationships, however this did not result in shared decision-making.

### Trust based on role perceptions

The level of trust was positively related to the extent to which roles and role boundaries were accepted. This was relatively straightforward in relationships between GPs and private sector allied health providers where the roles of the latter complement those of GPs and the TCAs limit the numbers of allied health consultations that can be reimbursed in any one year. There were more risks and uncertainties associated with collaboration between general practice and the diabetes centre, which in part was to do with an overlap in their roles. Both groups expressed strong identification with having a central role in diabetes care. Improvements in the quality and capacity of some general practices meant they were shifting into areas previously the domain of more specialist diabetes services. This is at times a complex and unpredictable environment with considerable variation in the levels of trust and mistrust. Some GPs reported a reluctance to refer patients to the diabetes service as: *"...they take over their care."(GP 1)*, although another GP reported that *"They don't pinch clients, they don't take them over, they always send them back." (GP 4) *These different perceptions illustrate the subjective nature of trust.

The increasing prevalence of T2DM and workforce shortages also influenced perceptions of vulnerability and risk. In response to the volume of work, one specialist medical practitioner was shifting routine follow up and screening to an allied health profession, whilst acknowledging that:

"..people will protect their income.... and if you're working really hard then you're not actually trying to do that, you're just trying to get through the workload. If you didn't have anything to do then you'd want to get people back." (Medical specialist 2)

### Trust is based on demonstrated competence

Respect and trust were intertwined and related to a mix of professional and personal factors. Recognising that other health professionals have complementary skills and competencies which can enhance and not duplicate care was a necessary precondition of respect and a willingness to collaborate, as the following quote illustrates:

*"I believe that good primary care requires collaboration. I don't hold either the knowledge or the mortgage on the right ways to support patients." (GP 3)*.

The second aspect was confidence in the competency of other professionals. For some health professionals, this was demonstrated through the quality of referral feedback information and patient feedback. GPs in particular judged the competency of allied health professionals from the relevance of the information contained in their reports and the timeliness of their communication. GPs preferred to refer patients to those practitioners who met these expectations. Allied health professionals who relied on TCA for an increasing proportion of their business recognised this and responded accordingly.

Confidence in other health professionals reduced the uncertainty of collaborating. A tacit knowledge and understanding of how other health professionals worked helped to reduce this uncertainty. Greater effort was required to establish and maintain relationships where the ground rules were not well known, or where there were different expectations.

"I find with the private guys I send people to I usually get information back quite quickly and know exactly what's been covered, and if there are any gaps I need to fill. I feel like it's quite a team approach. Whereas with the diabetes educators and others at the area health service... they don't often give me a great deal of information about what they're actually doing with people." (GP 5)

This quote also highlights the relationship between confidence and communication in the development of trust.

### Trust develops over time with good communication

Direct communication, usually by telephone, provided opportunities for the development of rapport, respect and trust in ways not afforded by referral letters and feedback reports. This, however, depended on the nature and tone of the communication. Communication characterised by a lack of respect could have the opposite effect. The receptivity and responsiveness of other health professionals was an important indicator of the quality of the relationship, mutual respect and trust. The following quotes illustrate the variation that existed:

" We generally have a good rapport with the referring doctors and we are able to contact them easily and discuss things if need be. I think that rapport has been built up over many years of treating patients and them getting to know us and what we do." (Dietician 2)

"...when we do have problems and we write to the GP, for example asking them to review a wound, we often don't hear back. That's very frustrating!" (Community nurse 2)

Opportunities for social interaction and the development of personal relationships help to foster trust and respect [10]. The rural context was a major enabler and most health professionals knew one another through a web of personal and professional linkages. Interprofessional and interdisciplinary education and training activities were important ways for different practitioners to come together and learn more about each other's roles, contributions and ways of working.

"I think what we've found is that the practice nurses have been a real bonus.... They've worked well with us and quite a lot of them have come to the diabetes course we run and we've got to know them, build up the rapport and they're comfortable about ringing us about anything." (Diabetes educator 2)

### Patient experiences

The effect of power and trust relations between health professionals on patient experiences related to their access to health services and the continuity of care they received from multiple providers.

#### Access to health services

Most patients recalled being referred by their GP to the diabetes centre for education when they were first diagnosed and for stabilisation when first commencing insulin. This finding indicates that despite the reservations expressed by GPs about the diabetes centre, they acknowledged the centre's role in these two areas. Patients who reported being on MDC plans were receiving care from a broad range of allied health professionals many of whom they had never or seldom seen before. This contrasted with other patients, not on these plans, who were referred to few allied health services, suggesting that these GPs were less willing to collaborate. The preference of GPs to refer patients to private sector allied health professionals was also evident in patient experiences.

"I get my eyes checked every 12 months, I've been to a podiatrist for my feet and I did a 10 week program with a dietician and exercise person at a health clinic that they run at a local gym." (Patient 5, aged 67)

#### Continuity of care

Aspects of continuity included timely communication and information exchange which facilitated consistent and complementary care over time. Patients' experiences varied. Patients who were not on MDC plans and TCA experienced a less connected and continuous form of primary health care, with each health professional providing their own care with little interprofessional communication and information sharing that patients were aware of. It was left to the patient as they saw fit to inform their main health provider of other care they were receiving. Patients on MDC plans and TCA experienced a greater continuity of care. The care plan was identified by patients as the common focus of consultations by the multiple providers. The reporting back by allied health professionals to the referring GP facilitated information sharing and consistency of care.

"the podiatrist sends a report after each meeting, and the practice nurse told me she'd entered it into the computer. (Patient 8, aged 65)

Patients who required a higher intensity of collaboration when their diabetes was unstable spoke about the direct two-way communication between the various health professionals during this short term period of more intensive management. This suggests that despite a lack of trust between GPs and the diabetes centre, their concerns about patients could override their disinclination to collaborate.

"I can't think why I went to the diabetes educator, but my readings were up and she suggested I see my GP and that I might need to go on insulin. She contacted him and he started me off on insulin. And because I started on insulin I also went to see the medical specialist at the diabetes centre as well and my GP was conversing with him and they agreed that it was important to see both Drs at that time." (Patient 3, aged 67)

## Discussion

This case study in a rural setting examined how power dynamics and trust influence interprofessional relations and how they impact on patient experiences. Three themes emerged in relation to power dynamics: (a) the use of professional power to protect autonomy; (b) power dynamics between private and public sector providers; and (c) reducing professional dependency to maintain power. The findings are consistent with earlier research conducted when the CDM program was introduced [5,7]. Despite the intention of the policy to support more shared decision-making, after three years, there is little evidence that this is happening. GPs maintained their authority by engaging in a low level of collaboration with allied health professionals. Tensions between GPs and public sector services over referral criteria have been identified in previous research [6]. That research found that secondary services based their decisions more on their internal capacity and roles than of the needs of GPs or the patients they wanted to refer. Our findings suggest that these tensions can be explained by power dynamics and conflicts over who has the authority to make referral decisions. The potential threats to independence and autonomy were more personal for health professionals than the organisational costs of negotiating around cultural and structural differences [30]. A comparative case study on health care networks likewise found an interaction between interorganisational, interprofessional and intraprofessional relationships that were to do with shifts to the balance of power, especially professional hierarchies and traditional power relations, or what has been called the 'dark side' of organisational relationships [31]. Our findings highlight the need to recognise the challenges in achieving more shared planning and decision-making between health professionals as this involves changing the power dynamics.

The major themes that emerged relating to trust were: (a) trust was based on role perceptions; (b) trust was also based on demonstrated competence; and (c) trust developed over time with good communication. The latter two themes are consistent with previous research that examined nurse-doctor relationships in primary health care settings [22]. Our study builds on that research by identifying the interaction between trust and role perceptions that goes beyond understanding each other's roles and professional identity. The delivery of primary and community-based health services that crosses organisational boundaries adds a layer of complexity to interprofessional relationships. The roles of, and role boundaries between and within, professional groups and services are changing. The uncertainty and vulnerability associated with these changes has affected the level of trust and mistrust, particularly between general practice and public sector community health services.

The findings confirm that trust and respect between different professions and disciplines facilitates cooperation. Unlike the difficulties in shifting the balance of power in interprofessional relationships, trust and respect can be fostered through a mix of interventions aimed at building personal relationships and establishing agreed rules that govern collaborative care and that are perceived as fair. The focus of most attention to date has been the private sector; extending these interventions to private and public sector relationships, clarifying the roles and contribution of each sector and addressing the concerns of the various groups is the next step.

Power dynamics and trust affected the strategic choices made by each health professional about whether to collaborate, with whom, and to what level. These decisions directly influenced patient experiences. Patients on MDC plans and TCA received more coordinated care than other patients and, consistent with other research, more comprehensive care [32]. For other patients, however, where there was less commitment by their main health care provider to collaborative care, there was not the same level of access to other health professionals. Collaborative arrangements were more informal and there was less continuity or connectedness of care and it was left to patients to take on the coordination role.

Together resource dependency and transaction cost analysis theories provide further insight of factors influencing interprofessional collaboration than either alone and point to the need for an integrated theory. The emphasis on transaction costs is particularly pertinent under fee-for-service funding models where only direct service provision activities are funded. Government policies have been mostly focussed at subsidising some of the additional costs associated with communication and information exchange amongst particularly private sector primary health care services. Referrals are a ubiquitous collaboration mechanism in the health field and the TCA can be viewed as a governance mechanism that regulates collaboration. However the use of referral mechanisms and TCA were inferior substitutes for trust when there were underlying power imbalances, conflicts and competition over resources (including clients and status).

This study has a number of limitations. The results are based on interviews in one rural setting and, although they are typical of such areas, the experiences may be different in urban areas where there is a less defined catchment population and a greater range and number of health professionals. Moreover, the experiences may not be the same in countries with different funding and organisational arrangements for primary and community health services to those found in Australia. Self-selection within the professional categories may have been biased towards practitioners with a commitment to collaboration. The number of patients was small and any with significant complications or late stage disease were excluded. The transferability of the findings for these and other patient sub-groups should be regarded with caution. Notwithstanding these caveats, sufficient description has been provided for readers to interpret the findings and consider their applicability for their circumstances.

## Conclusion

This research highlights the complex power dynamics associated with collaboration between different health professions and disciplines across organisational boundaries and the role played by trust in these relationships. MDC plans and TCA aim to increase multidisciplinary care and provide incentives for GPs and allied health professionals to collaborate with one another. Professional relations were a dominant factor that influenced how the policy was implemented at the micro level. Power dynamics and trust concepts enable us to understand what took place. Collaboration involves uncertainty and risk and challenged professional autonomy and independence to a greater or lesser extent. The groups whose authority and status was most challenged by recent policy initiatives to support more collaborative care were GPs and public sector practitioners. Some GPs passively resisted more shared-decision making with allied health professionals, and public sector practitioners resisted the incursion of GPs into areas where traditionally they had little authority or status. Trusting and respectful interprofessional relationships could lessen the threats and risks of collaboration. This combination of factors influenced the decisions by health professionals about whether to collaborate across organisational boundaries, with whom and to what level. The level of collaboration rarely went beyond a low level of interdependency, and together with the policy, GP preferences have seen a shift of primary and community-based care to the private sector and away from the public sector. The experiences of patients reflected the impact of these decisions.

## Competing interests

The authors declare that they have no competing interests.

## Authors' contributions

JM developed the study design, undertook the data collection, conducted the data analysis, and wrote the first draft of the manuscript. MH and RJ contributed to study design and contributed to the interpretation of the findings. All authors contributed to the subsequent manuscript drafts and approved the final manuscript.

## Pre-publication history

The pre-publication history for this paper can be accessed here:

http://www.biomedcentral.com/1472-6963/12/63/prepub
